# A Combined Epigenetic and Non-Genetic Approach for Reprogramming Human Somatic Cells

**DOI:** 10.1371/journal.pone.0012297

**Published:** 2010-08-19

**Authors:** Jinnuo Han, Perminder S. Sachdev, Kuldip S. Sidhu

**Affiliations:** Faculty of Medicine, University of New South Wales, Sydney, Australia; City of Hope Comprehensive Cancer Center, United States of America

## Abstract

Reprogramming of somatic cells to different extents has been reported using different methods. However, this is normally accompanied by the use of exogenous materials, and the overall reprogramming efficiency has been low. Chemicals and small molecules have been used to improve the reprogramming process during somatic cell nuclear transfer (SCNT) and induced pluripotent stem (iPS) cell generation. We report here the first application of a combined epigenetic and non-genetic approach for reprogramming somatic cells, i.e., DNA methyltransferase (DNMT) and histone deacetylase (HDAC) inhibitors, and human embryonic stem cell (hESC) extracts. When somatic cells were pretreated with these inhibitors before exposure to hESC (MEL1) extracts, morphological analysis revealed a higher rate of hESC-like colony formation than without pretreatment. Quantitative PCR (qPCR) demonstrated that pluripotency genes were upregulated when compared to those of somatic cells or treated with hESC extracts alone. Overall changes in methylation and acetylation levels of pretreated somatic cells suggests that epigenetic states of the cells have an effect on reprogramming efficiency induced by hESC extracts. KnockOutserum replacement (KOSR™) medium (KO-SR) played a positive role in inducing expression of the pluripotency genes. hESC extracts could be an alternative approach to reprogram somatic cells without introducing exogenous materials. The epigenetic pre-treatment of somatic cells could be used to improve the efficiency of reprogramming process. Under differentiation conditions, the reprogrammed cells exhibited differentiation ability into neurons suggesting that, although fully reprogramming was not achieved, the cells could be transdifferentiated after reprogramming.

## Introduction

Currently, there are four different strategies used to reprogram somatic cells: i) somatic cell nuclear transfer (SCNT) [1], ii) transduction of pluripotent genes into somatic cells [2], iii) somatic cell fusion with pluripotent cells [3], and iv) pluripotent cell extract mediated de-differentiation [4]. While SCNT and iPS cells have drawn much attention, somatic cell reprogramming induced by fusion with ESCs and by exposure to pluripotent cell extracts has not been well studied.

The mechanism of reprogramming is not clear. However, epigenetic changes have been known to be important as both global and gene-specific DNA and histone modifications have been observed in reprogramming *in vitro* [5]. DNA methylation status of genes promoter regions is associated with transcriptional activities [6] and research has shown that mouse ESC genomes are less methylated than those of somatic cells [7], [8]. In human, it has also been shown that hESCs have a distinct epigenetic signature from somatic cells [9]. Higher levels of histone acetylation are found in pluripotent cells than in somatic cells [10]. Acetylation of H3 at Lysine 9 (H3K9) has been recognized as one of the most important epigenetic markers, which, when abundant in the promoter region of genes, represent an active status and is correlated with gene expression [11], [12].

DNA methylation is known to be catalyzed by DNMTs [13], while histone deacetylation is catalyzed by HDACs [14]. Inhibitors of these enzymes have been used in reprogramming experiments. One of the DNMT inhibitors, 5-aza-2′-deoxycytosine (5-aza-dC) has been shown to silence imprinted gene expression in mouse somatic cells by decreasing DNA methylation levels [15] and others have used this demethylating agent to improve SCNT [16] and iPS cell generation [17]. Similarly, when a HDAC inhibitor, Trichostatin A (TSA) was applied to somatic cells, improvement in nuclear cloning and iPS cell generation were also reported [18], [19]. All-trans retinoic acid (ATRA) is known to bind to RA receptors and activate Histone acetyltransferases (HAT) thus acts as an indirect inhibitor of HDAC. It was demonstrated to induce nucleosomal repulsion, chromatin relaxation, gene transcription [20] and reduce cytosine methylation in of somatic cells [21].

Mouse embryonic stem cell (mESC) and the human embryonic carcinoma cell (ECC) extracts have shown to reprogram somatic cells to some extent [4], including reactivation of pluripotency genes [22], chromatin remodeling [23], engraftment and transdifferentiation of the reprogrammed cells *in vivo* [24]. However, opposite results were also reported [25] and hESC extracts has not been tested for reprogramming somatic cells. Furthermore, no attempts to transform the knowledge obtained from other reprogramming approaches, such as applying small molecules to improve the event, has been reported. Thus we hypothesized application of the above DNMT and HDAC inhibitors to somatic cells, the chromosomes of the cells would decondense, and provide an easier access for reprogramming factors present in hESC extracts to function.

In the present study, we report for the first time that hESC extract induces reprogramming in human fetal fibroblasts (HFFs) as determined by morphological changes and re-activation of ESC specific makers. The reprogramming efficiency could be improved by pre-treatment with DNMT and HDAC inhibitors. Reprogrammed HFFs could be directly differentiated into DA neurons when co-cultured with PA6 stromal cells. This reprogramming approach without the use of gene transduction provides the possibility for future therapeutic application. Lastly, our studies have demonstrated that the p53/p21 pathway is activated during reprogramming process under the culture conditions used here and thus plays a negative role in hESC extract induced reprogramming.

## Results

### Morphological changes in HFFs induced by hESC extract treatment

**Figure 1:**
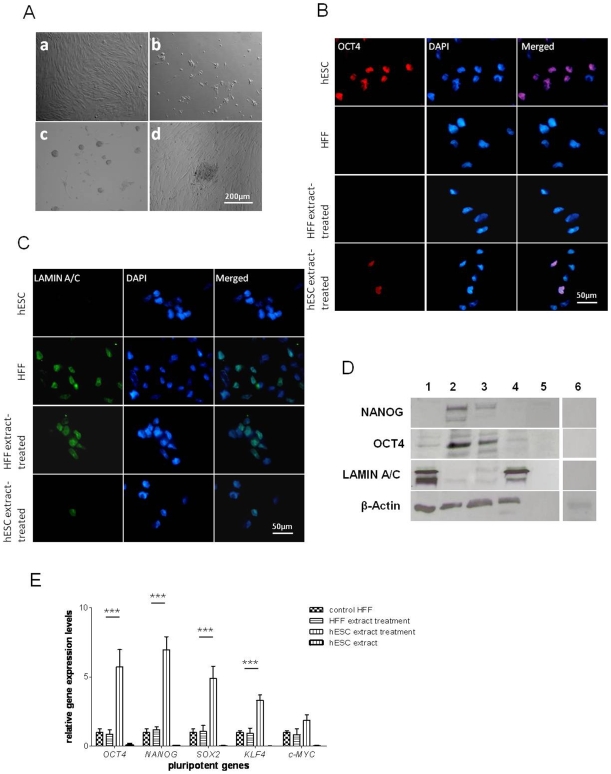
Morphological, protein and gene expression changes in HFFs induced by hESC extract treatment. (A) Morphology of HFFs treated with its own extract (a) or hESC extract on day 3 (b), day 10 (c) and passage 2 (d). (B) OCT4 expression was induced in hESC extract-treated HFFs on day 7. (C) LAMIN A/C was demolished in hESC extract-treated HFFs on day 7. (D) Imunoblotting analysis of NANOG, OCT4 and LAMIN A/C expression on day 7. Lanes from 1 to 6 were loaded with proteins (2 µg) from HFFs, hESCs, hESC extract and HFF extract-treated HFFs, water and hESC extracts. (E) Expression of *OCT4*, *NANOG*, *SOX2*, *KLF4* and *C-MYC* were upregulated in hESC extract-treated HFFs after 7 days of hESC extract treatment. The gene expression levels were normalized to the *GAPDH* and compared relative to gene expression in control HFFs. Error bar, S.D., ^***^P<0.001 (n = 3).

The membrane of HFFs were first permeabilized with Streptolysin-O (SLO). The cells were then exposed to either hESC or HFF extracts (control) obtained from same number of cells. After membrane resealing, the cells were cultured in mTeSR™1 medium for 3 days and remarkable morphological differences between treated and control samples were observed (**Figure 1A**). The control cells exhibited no morphological changes up to 14 days, whereas most of the hESC extract-treated cells demonstrated a rounded cell morphology, resembling hESCs, and started to form small clusters as early as 3 days post-treatment (**Figure 1A (b)**). When manually transferred to feeder cells, colonies with hESC-like morphology were formed (**Figure 1A (d)**). The efficiency of colony formation was 1.2±0.3×10^−4^% from 6 independent experiments. Colony formation was not observed among HFF extract-treated cells. To trace the origin of the putative reprogrammed cells derived from HFFs after hESC extract treatment and also to exclude the possibility of contamination from hESCs, DNA microsatellite markers were analyzed for HFFs, hESCs and hESC extract treated HFFs. As shown in **Table 1**, the patterns of 15 short tandem repeats were matched between hESC extract treated HFFs and parental HFFs, which differed from hESCs. In addition, STR analysis showed a male allele pattern for hESCs and female allele pattern for reprogrammed HFFs and parental HFFs.

**Table 1:** STR analysis of hESCs, HFFs and reprogrammed cells.

Locus	MEL1	HFF	Reprogrammed cells
**D8S1179**	13, 15	10, 14	10, 14
**D21S11**	30	27	27
**D7S820**	11, 12	8	8
**CSF1PO**	10, 14	10, 11	10, 11
**D3S1358**	15, 18	16	16
**TH01**	9.3	9	9
**D13S317**	11, 13	8, 9	8, 9
**D16S539**	12, 13	11, 13	11, 13
**D2S1338**	23	20, 25	20, 25
**D19S433**	14, 15	12, 16.2	12, 16.2
**vWA**	16	15, 18	15, 18
**TPOX**	8, 9	8	8
**D18S51**	12, 16	12, 13	12, 13
**D5S818**	11, 12	11, 12	11, 12
**FGA**	21	22	22

### Changes in pluripotent/differentiation marker expression patterns and epigenetic states in HFFs during hESC extract induced reprogramming

Immunoblotting and immunofluorescent staining were performed 7 days post hESC extract treatment. When hESC extracts were loaded on a SDS-PAGE gel, no protein could be detected to cross-react with β-actin, OCT4 or NANOG antibodies, indicating the absence of these proteins. Thus, any protein detected should be from the reprogrammed cells rather than from the hESC extracts itself. OCT4 is strongly expressed in >90% of hESCs but not in HFFs. Expression of OCT4 was detected in 23.3±4.3% of HFFs treated with hESC extracts after 7 days of hESC extract treatment (**Figure 1B**). NANOG remained undetectable in either hESC or HFF extract-treated HFFs on day 7 (**Figure S1**). In contrast, a differentiated cell marker LAMIN A/C was lost in 80.3±4.8% of HFFs after the hESC extract treatment (**Figure 1C**). These data were confirmed by immunoblotting analysis and NANOG protein band was also detected in hESC-extract treated HFFs 7 days post-treatment (**Figure 1D**), suggesting that HFFs were induced towards pluripotency while differentiated characteristics were lost. Next, we determined whether pluripotency-related genes *OCT4*, *NANOG*, *SOX2*, *C-MYC* and *KLF4* were transcriptionally induced by performing qPCR. As shown in **Figure 1E**, 7 days after hESC extract treatment, 3 to 7 fold increases of gene expression were detected in HFFs after exposure to hESC extracts. Under the same condition, no expression of the five genes was detected in hESC extracts.

**Figure 2:**
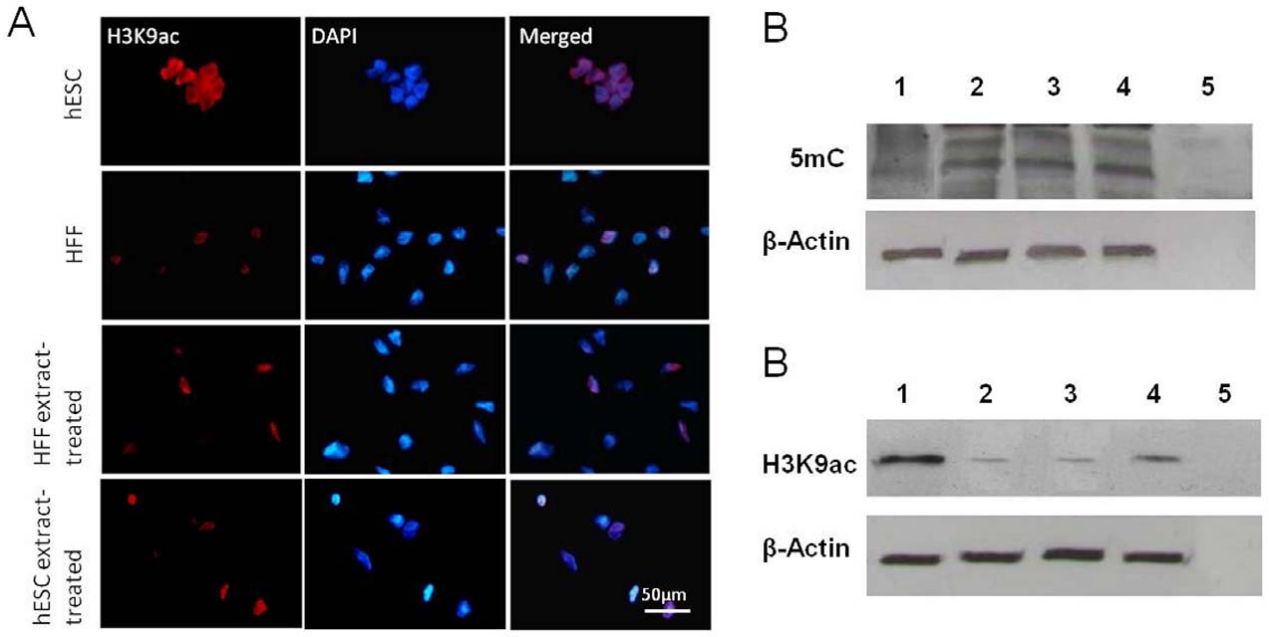
Global epigenetic changes in HFFs 7 days after hESC extract treatment. (A) Acetylation level of H3K9 was increased in HFFs after hESC extract treatment. (B) Immunoblotting analysis of 5-methyl cytosine. (C) Immunoblotting analysis of H3K9 acetylation levels. In A and C, lane 1 to 5 were loaded with proteins (2 µg) from hESCs, HFFs, HFFs treated with own extract or hESC extract, and negative control.

To determine whether this hESC extract induced reprogramming was mediated by epigenetic modification of somatic cell chromatin, DNA methylation and histone acetylation levels were examined. No changes in 5′-methylated cytosine (5 mC) in the nucleoplasm were observed between hESC extract-treated and non-treated HFFs (**Figure S2**). However, global DNA methylation was found to be slightly lower in hESCs than HFFs (**Figure 2B**). Global level of H3K9 acetylation in HFF nuclei was increased by hESC extract treatment. As **Figure 2A** shows, more than 90% of hESCs stained positively for histone H3K9, while a smaller fraction of HFFs (22.9±5.1%) were positively labeled, albeit with a weaker signal. This was not altered by exposure of HFFs to its own extracts; however, acetylation of histone H3K9 was restored after incubation with hESC extracts and 43.1±9.3% of the total cells were positive for H3K9. This increase in acetylation levels in hESC extract treated HFFs was further confirmed by immunoblotting analysis (**Figure 2C**).

### Demethylation of OCT4 and NANOG promoters and up-regulation of pluripotency-related genes in HFFs induced by DNMT and HDAC inhibitors

To evaluate the effect of DNMT and HDAC inhibitors on HFF nuclear re-modelling, 1 µM 5-aza-dC, 0.5 µM TSA and 0.1 µM ATRA were supplemented in F-DMEM, KO-SR or DMEM medium and cultured for 3 days. These concentrations of 5-aza-dC, TSA and ATRA did not induce significant cell death or inhibition of cell growth (**Table S1**). We examined the methylation states around the promoter regions of five pluripotency-related genes (*OCT4*, *NANOG*, *SOX2*, *C-MYC* and *KLF4*) by performing bisulfite sequencing analysis.

**Figure 3:**
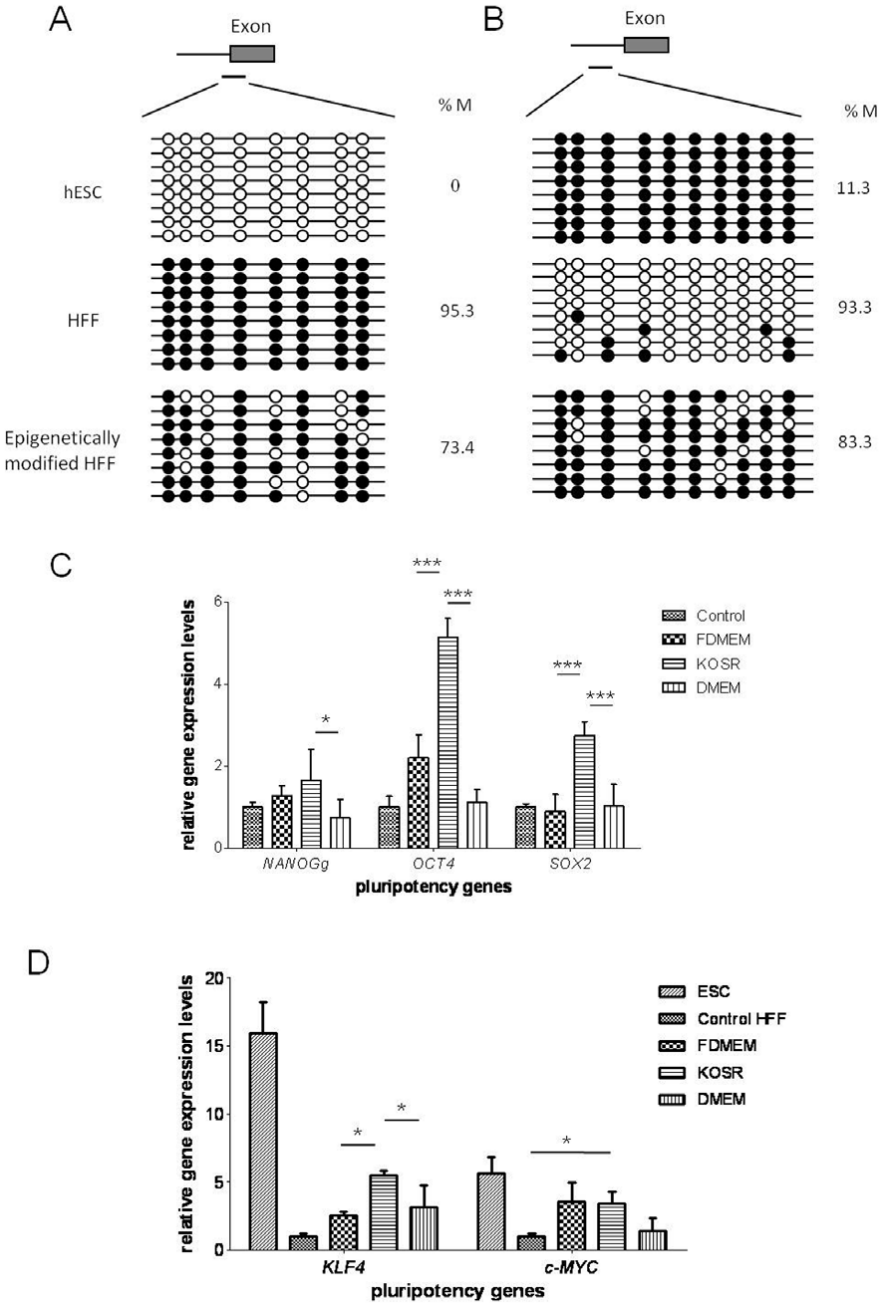
Effects of epigenetic modifications on HFFs. (A) Bisulfite sequencing of *NANOG* and (B) *OCT4* promoter region. Black circles represent methylated sites, white circles represent unmethylated sites. Global methylated cytosines are shown as %M. (C) qPCR analysis of *NANOG*, *OCT4* and *SOX2* and (D) *KLF4* and *c-MYC* expression in controls and HFFs with chemical treatment in different culture medium. The gene expression levels were normalized to the *GAPDH* and compared relative to gene expression in control HFFs. Error bar, S.D., ^*^P<0.05, ^***^P<0.001 (n = 3).

As shown in **Figure 3A**, The *NANOG* promoter region was highly methylated in HFFs (95.3%) and completely unmethylated in hESCs. A similar methylation pattern was observed in *OCT4* examined region, where 93.3% of the CpG sites were methylated in HFFs but only 11.3% were methylated in hESCs (**Figure 3B**). DNMT and HDAC inhibitor treatment was shown to decrease the methylation levels of both regions (73.4% and 83.3%, respectively). When methylation levels in CpG islands of *SOX2*, *c-MYC* and *KLF4* were tested, the overall CpG methylation levels were found to be lower than 5% for all three genes (**Figure S3**), indicating that their expressions are not regulated through DNA methylation.

To gain further insight into the changes in pluripotency-related gene expression patterns after the inhibitor treatment (72 hours), qPCR was performed. When 5-aza-dC or TSA were applied, pluripotency genes were not induced in HFFs (data not shown). However, as shown in **Figure 3C and 3D**, *OCT4*, *SOX2*, *c-MYC* and *KLF4* was upregulated up to 6 fold in 5-aza-dC, TSA and ATRA treated HFFs when compared to non-treated controls (P<0.05). Interestingly, medium components were shown to affect the induction of the genes in HFFs. Expression of *OCT4*, *SOX2* and *KLF4* was significantly higher in HFFs cultured in KO-SR medium than in F-DMEM medium (P<0.05), except *c-MYC*, the expression of the genes was higher in KO-SR culture than in DMEM culture (P<0.05).

### Morphological changes in HFFs epigenetically modified prior to hESC extract treatment

**Figure 4:**
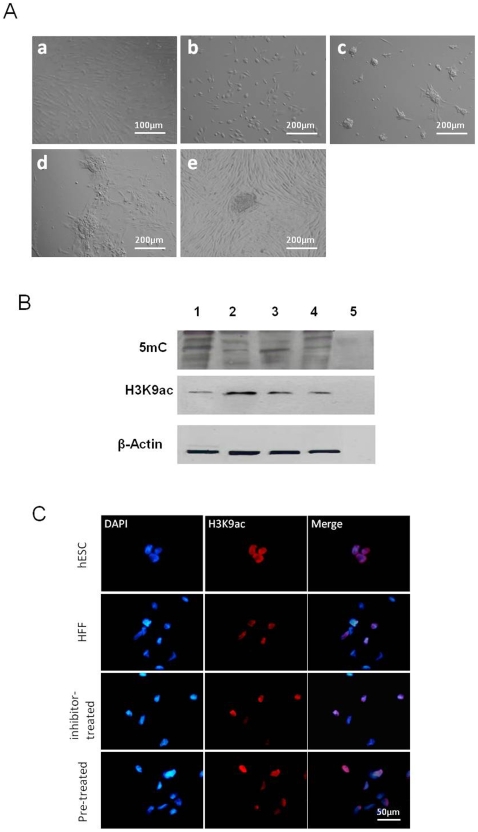
Morphological and epigenetic changes of HFFs after inhibitor and/or hESC extract treatment. (A) Morphological changes of HFFs elicited by DNMT and HDAC inhibitors and hESC extracts. HFFs in mTeSR (a) or with inhibitor treatment prior to hESC extract exposure on day 3 (b), day 7 (c), day 10 (d) and at passage 2 (e). (B) Immunoblotting analysis shows decrease in 5-methyl cytosine and increase in H3K9 acetylation levels after combined treatment. Lanes 1 to 5 were loaded with proteins (2 µg) from HFFs, hESCs, inhibitor treated HFFs and combined treated HFFs. (C) Immunostaining analysis shows increased H3K9 acetylation levels in HFFs after DNMT/HDAC inhibitor or combined treatment.

As early as 24 h after DNMT and HDAC inhibitor and hESC extract combined treatment, differences in cell morphology could be observed between control and treated HFFs. Without hESC extract treatment, epigenetically modified HFFs in mTeSR™1 medium were spread out and exhibited characteristic fibroblast morphology, whereas cells treated with the inhibitors and hESC extracts appeared to be short and flattened, without cell-cell contact (**Figure 4A (a)** and **(b)**). By day 3 (**Figure 4A (c)**), cell clusters formed in the culture at an efficiency of 0.017±0.009% (from 3 independent experiments). The clusters increased in size, and by 7 days post-treatment larger stem cell-like colonies were formed (**Figure 4A (d)**). When these cells were manually dissected and transferred to HFF feeder cells and cultured in KO-SR medium, colonies resembling hESCs (**Figure 4A (e)**) were formed at an efficiency of 1.35 to 2.07×10^−4^% (from 5 independent experiments).

### Global epigenetic changes and expression of pluripotency-related markers after combined treatment

To determine the overall methylation and acetylation status of HFFs after DNMT/HDAC inhibitor and hESC extract treatment, immunofluorescent staining and immunoblotting were performed one week later. There was no change in DNA methylation level in HFFs that were exposed to the inhibitors with or without post hESC extract treatment (**Figure S4**) On the contrary, the overall acetylation levels were increased (**Figure 4B**). As shown in **Figure 4C**, 52±4.5% and 67±5.9% of HFFs displayed intranuclear acetyl-histone H3 after chemical treatment and combined treatment, respectively. Whereas there were less HFFs positive for acetyl-histone H3 (32±7.3%) with the labelling signal at a lower intensity when compared to hESCs in which more than 90% of the cells were positively stained. This suggested a mild acetylation effect induced by the chemicals and/or hESC extract. However, none of the treatment was able to significantly increased histone acetylation level in HFFs (P<0.05).

**Figure 5:**
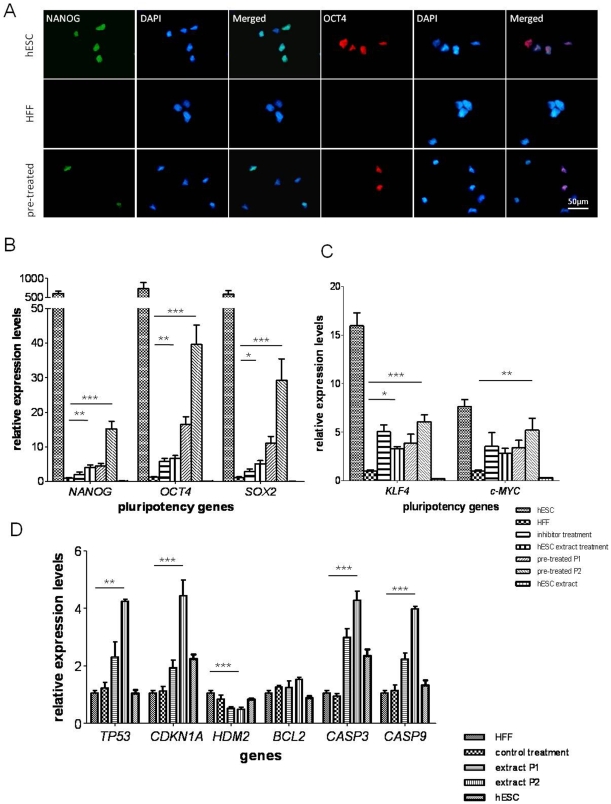
Protein and gene expression changes of HFFs after reprogramming treatment. (A) Pluripotency marker NANOG and OCT4 were induced in HFFs after reprogramming. (B), (C) and (D) qPCR analysis of pluripotency-related gene and apoptotic gene expression in controls and reprogrammed HFFs. The gene expression levels were normalized to the *GAPDH* and compared relative to gene expression in control HFFs. Error bar, S.D., ^*^P<0.05, ^***^P<0.001 (n = 3).

**Figure 5A** shows >90% of hESCs were labeled positively for NANOG and OCT4. In contrast, these two proteins were undetectable in somatic HFFs. However, the labeling for both transcription factors were obtained in HFFs after epigenetic modification followed by hESC extract treatment. Additionally, after 3 independent experiments we observed that 15±2.8% and 31±3.2% cells positively expressed NANOG and OCT4, respectively.

The expression levels of pluripotency-related genes were examined by qPCR 7 days post reprogramming treatment. *NANOG*, *OCT4* and *SOX2* were more than 500 fold higher in hESCs than in HFFs (**Figure 5B and 5C**), whereas *c-MYC* and *KLF4* were expressed 16 and 8 times higher in hESCs than HFFs, respectively. These transcripts were not detected in hESC extracts, confirming that the origin of the mRNAs was the cells rather than the extracts. The trend of pluripotent gene reactivation was observed after either chemical or hESC extract treatment alone. However, it was only through the application of a combined treatment that the genes: *NANOG*, *OCT4*, *SOX2* and *KLF4* resulted in a significant upregulation (P<0.05). Interestingly, passaging of the reprogrammed HFFs onto feeder layers seemed to play an important role in improving reprogramming, since P2 cells showed significant higher expression of all five tested genes than cells at P1 on matrigel (P<0.001). *NANOG*, *OCT4* and *SOX2* were expressed 15 to 40 fold higher in P2 cells than in control, while *c-MYC* and *KLF4* expression increased by 8 fold.

### Activation of p53/p21 pathway in HFFs induced by chemical and hESC extract treatments

Reprogramming is now known to be a stress process [26], and the low efficiency of hESC extract induced reprogramming in the current study seemed to support this point of view. Thus, it was hypothesized that p53/p21 apoptotic pathway plays a negative role in cell growth and self-renewal in hESC extract induced reprogramming. As shown in **Figure 5D**, control HFFs exposed to their own cell extracts did not shown changes in expression level of the apoptotsis-related genes. However, after reprogramming and passaging onto irradiated feeder cells, hESC extract treated cells at P2 exhibited significantly higher expression levels of *TP53* and *CDKN 1A* compared to those of control HFFs (4 fold, P<0.05). The expression of *HDM2* was significantly lower than that in control HFFs, whereas no difference of anti-apoptotic *BCL2* expression was observed between reprogrammed cells and control HFFs. *CASP3* and *CASP9* were expressed at two and four fold higher in reprogrammed HFFs than those in control cells. Coincidently, upon passaging of the reprogrammed cells, a further upregulation of *TP53*, *CDKN1A*, *CASP3* and *CASP9* was observed, which may have resulted in the cells inability to proliferate further.

### Directed differentiation of reprogrammed HFFs into neuronal cells

**Figure 6:**
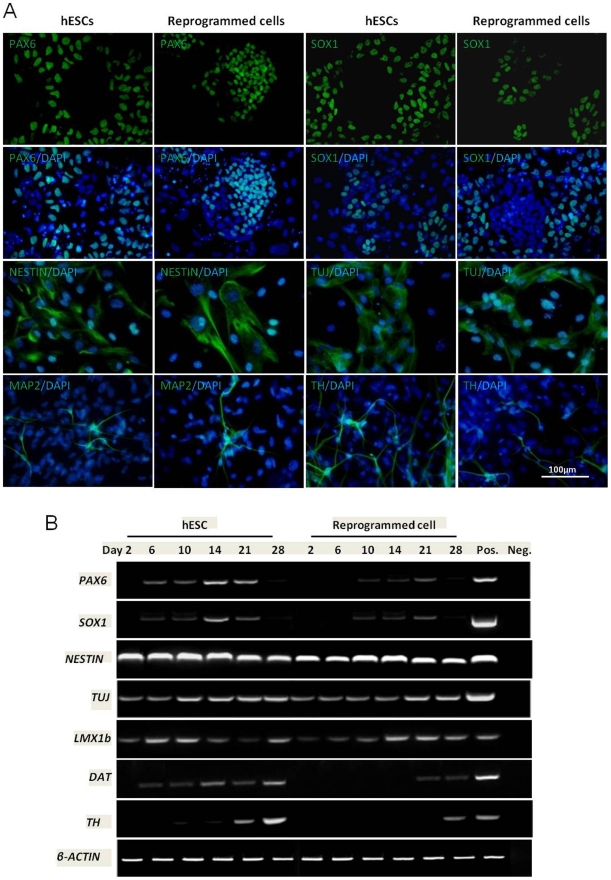
Expression of neuronal markers upon directed differentiation. (A) immunostaining of protein expression (B) RT-PCR analysis of mRNA expression in hESCs and reprogrammed cells during the differentiation process.

We next determined the differentiation capacity of these reprogrammed cells into a neural lineage. The reprogrammed fibroblasts were co-cultured on a monolayer of stromal cell-derived inducing activity (SDIA) cells, PA6. The neuronal differentiation was analyzed by examining morphological changes as well as expression of neuronal specific genes and proteins. Morphological appearance of the cells indicated the formation of neural lineages upon directed differentiation. Furthermore, as shown in **Figure 6A**, these reprogrammed cells acquired protein expression specific to neuronal stem cells, neuronal precursors, immature neurons, mature neurons, early DA neurons and also mature DA neurons. PAX6 and SOX1 were detectable in differentiated cells (both hESCs and reprogrammed) on day 7; NESTIN and TUJ started to be expressed by differentiating cells from day 7 but the density peaked on day 14. More mature neuronal makers MAP2 and TH were only stained after 3 weeks of differentiation.

Expression of neuronal marker genes in the differentiating cells was analyzed by RT-PCR (**Figure 6B**). *PAX6* was first detected on day 6 in hESCs and on day 10 for reprogrammed HFFs. There was a slight increase in this neural precursor gene during the two weeks of differentiation and diminished by week four. Similarly, definitive neural marker *SOX1* showed a similar trend. However, NESTIN was expressed during the whole neural differentiation (as well as in undifferentiated hESCs). Similarly, *TUJ* and *LMX1b* were also detected through the entire differentiation process. DA neuron markers *DAT* and *TH* were expressed in differentiated hESCs on day 10 and day 21, respectively. However, in reprogrammed cells, *DAT* expression was only detectable from day 21 and at a low level. Delayed expression of *TH* was also observed in reprogrammed HFFs, which was later expressed from day 28.

## Discussion

The ultimate goal of reprogramming is to generate isogenic pluripotent stem cells derived from adult somatic cells, so that there is no graft rejection during transplantation into the host. Extracts from pluripotent cells (i.e. mESCs and hECCs), *Xenopus* [27], [28] and mammalian [29] oocytes have been used to induce somatic cell nuclear reprogramming [4], [22], [23], [24], [30]. However, there has been no report of using hESC extracts for the same purpose. In the current study, we demonstrated that human somatic cells can be reprogrammed to a stem cell-like state by a combined treatment with epigenetic modifying reagents (DNMT and HDAC inhibitors) and hESC extracts. This was evidenced by morphological changes, epigenetic changes and expression of pluripotency-related genes and proteins during and after reprogramming.

Morphological changes in the somatic cells transiently exposed to hESC extracts were observed as early as 24 h post-treatment. The formation of stem cell-like colonies was observed 10–14 days post-treatment, which was similar to the time needed for transcription factor-induced reprogramming [31]. Upregulation of pluripotency markers and downregulation of differentiation markers are indicators of somatic cell reprogramming. *Oct4* is required for pluripotency and reduction of *Oct4* expression below 50% induces differentiation [32]. After incubation with hESC extracts, a small percentage of somatic cells expressed OCT4, indicating their dedifferentiation towards an embryonic state. At the same time, LAMIN A/C expression, a marker of differentiated cells [33] was decreased. Similar expression patterns for pluripotent and differentiation genes were observed when somatic cells were exposed to NCCIT, mESC or *Xenopus* oocyte extracts [24], [30], [31]. However, in those studies, more than 50% of the total somatic cells were reported to express the OCT4 protein, whereas in the current study less than 30% of the treated HFFs were positive for OCT4. This could be due to the difference between extracts from different types of cells. The qPCR data confirmed the upregulation of pluripotency associated genes, i.e. *OCT4, SOX2*, *c-MYC* and *KLF4* after hESC extract treatment, affirming that the upregulation of these genes were made by components in the hESC extracts, but not by the extract treatment.

Previous studies have demonstrated specific-gene promoter demethylation and acetylation of *Oct4* and *Nanog*, in somatic cells after mouse ESC or human ECC extract treatment [23], [24]. In our study, we detected genome wide changes of demethylation and acetylation induced by hESC extracts. These changes raise the possibility that components in the hESC extracts may have similar effects to other pluripotent cell extracts on modifying the epigenetic status of somatic cells.

*Oct4* and *Nanog* expressions are known to be regulated by epigenetic mechanisms and can be altered by 5-aza-dC or TSA [34], [35]. While *Oct4* was reported to be expressed in somatic cells only when 5-aza-dC and TSA were applied, *Nanog* was unable to be reactivated by these two chemicals in TS cells [34], [35]. However, a more recent research reported that 5-aza-dC and TSA are able to up regulate *Oct4*, *Nanog* and *Klf4* in neurospheres [36]. We found that neither DNMT nor HDAC inhibitor alone is sufficient to increase the expression of pluripotency genes in HFFs (data not shown). However, with the combination of 5-aza-dC, TSA and ATRA, pluripotency-related genes were upregulated compared to those in non-treated HFFs. Interestingly we found a relationship between gene expression levels during reprogramming in somatic cells and the culture media. The reason for this medium effect is not known, however, the commercially purchased KnockOut™ serum replacement (KOSR™) (Invitrogen) is a product with more-defined growth supplement (exact formulation is not described) that reduces spontaneous differentiation in ESCs. It has been reported that FBS-containing medium does not support hESC growth whereas KO-SR medium is able to support extended hESC growth [37]. KOSR™ may also promote reprogramming as suggested by previous reports showing that compare to FBS, KOSR™ can improve embryonic stem cell-line derivation and iPSC generation [38], [39]. Our bisulfite sequencing analysis revealed partial demethylation of *NANOG* and *OCT4* promoter regions in HFFs after DNMT and HDAC inhibitor treatment, which are consistent with previous reports [34], [35]. The methylation status in *OCT4* and *NANOG* promoter regions together with the gene expression patterns indicated that the HFFs were undergoing an event towards a more naïve state, which may explain the reason why hESC culture medium is more suitable for reactivating pluripotency-related genes.

We then combined epigenetic modification of HFFs with hESC extract treatments and more defined evidence of reprogramming was observed. As per **Figure 4**, reprogramming efficiency, colony formation, pluripotent-associatedprotein expression indicated that pre-treatment with epigenetic modifying agents of somatic cells is beneficial for hESC extract-mediated reprogramming. The pluripotency-related genes in reprogrammed HFFs were also expressed at higher levels than epigenetically modified HFFs or hESC extract-treated HFFs alone. In a recent report, where mouse ESC extracts were used to induce somatic cell reprogramming, researchers were not able to detect *Oct4* expression until 6 weeks after treatment [30]. This could be another sign of advantage of applying epigenetic modifying agents before inducing reprogramming, since both OCT4 and NANOG were detected 5 days post-treatment in the current study.

The ability to self-renew indefinitely is one of the most defining characteristics of ESCs. However, proliferation and self-renewal properties of reprogrammed cells in this study were not comparable with hESCs. The proliferation rate of colonies generated was slower than hESCs and they could not be maintained in culture with typical hESC morphology for extended periods. Since reprogramming is a stressful process, it is possible that the p53/p21 apoptotic pathway could play a negative role in cell growth and self-renewal. Several groups have reported that p53/p21 pathway serves as a barrier in nuclear reprogramming during iPS cell generation [26], [40], [41], [42]. As shown in **Figure 5D**, there was a considerable increase in *TP53* and *CDKN1A* expressions in reprogrammed cells suggestive of p53/p21 pathway activation, which may be induced by reprogramming and DNA damage. Downstream genes of *TP53* that are involved in cell apoptosis (*CASP3* and *CASP9*) [43] were also induced by reprogramming treatment. *HDM2* is a regulator of p53 and mimicking p53 suppression in reprogramming [44]. Decreased *HDM2* levels further confirmed the negative effect of p53/p21 pathway on reprogramming. Although the mechanism of p53 activation in the reprogramming process is not clear, the initial stress generated by chemicals and hESC extracts to induce reprogramming can be a crucial one. Upregulation of apoptotic genes after reprogramming in HFFs strongly support the possibility that p53-dependent apoptosis is the main factor decreasing cell viability and loss of self-renewal in the reprogrammed HFFs. A more apparent explanation would be that the reprogrammed cells have resided at an intermediate stage and have not fully gained hESC properties since the expression levels of pluripotency-related genes were significantly lower compared to hESCs. Recent applications of the ROCK inhibitor Y-27632 in enhancing survival of hESCs and generating iPS cells provide the possibility to circumvent this problem [45], [46]. However, the reprogrammed cells were able to directly differentiate into neuronal cells when co-cultured with PA6 cells. This testified the differentiation ability of reprogrammed cells and suggested that lineage reprogramming may have took place since complete reversion to pluripotency did not occur. Most recently it was reported that by manipulating culture conditions, endogenous expression of stem cell genes were induced in somatic cells, but it was a short-term induction [47]. Similarly, by changing the microenvironment (applying ESC conditional medium and epigenetic modifying molecules), iPS cells were generated from rat progenitors [48]. Our data provide an alternative method of reprogramming without introduction of genetic materials or exogenous factors into somatic cells.

In summary, our study demonstrated that components of hESC extract can modify the chromatin of HFFs and this nuclear remodeling leads to reactivation of pluripotency-related genes and repression of differentiation markers. Reprogramming can be promoted by pre-treatment with DNMT and HDAC inhibitors, which function through epigenetic modifications of the somatic genome and the resulting cells possess the differentiation capacity under appropriate conditions. However, upon extended culturing, reprogrammed cells could not be maintained, most probably due to p53/p21 pathway activation, which may function as a negative regulator of the reprogramming process. Our research provides a way of studying the features of reprogramming and the possibility of identifying factors involved in reprogramming by analyzing the components of the hESC extract. Application of small molecules and hESC extracts may lead to efficient reprogramming without altering the somatic genome. This approach also demonstrates that in order to obtain a certain type of cell, it is not necessary to revert cells back to a pluripotent state, followed by differentiation. This approach to reprogramming provides a potential source of patient-specific cells with possible application in regenerative medicine.

## Methods

### Cells and cell culture

hESC line, MEL1 (CHEMICON, Millipore, USA) was maintained on γ-irradiated human fetal fibroblasts (HFFs) and cultured in serum replacement (SR) medium, containing KO-DMEM-high glucose, 20% SR, 2 mM L-glutamax, 0.1 mM non-essential amino acid, 0.1 mM 2-mercaptoethanol, 1× insulin transferring selenium, 25 U/ml penicillin, 25 µg/ml streptomycin and 4 ng/ml basic fibroblast growth factor (bFGF). hESCs were subcultured every 5–7 days using 0.05% Trypsin. HFFs were derived from primary fetal skin tissue after therapeutic termination of pregnancies and cultured in DMEM-high glucose medium containing 10% fetal bovine serum (FBS), 25 U/ml Penicillin and 25 µg/ml Streptomycin. All reagents were from Invitrogen unless otherwise stated.

For demethylating and acetylating treatment, P4 to P6 HFFs were pre-cultured in F-DMEM to about 50% confluence before being cultured in F-DMEM, KO-SR or DMEM medium containing 1 µM 5-aza, 0.5 µM TSA and 0.1 µM ATRA for 72 hours or kept untreated for 3 days. Cell viability testing was performed using fluorescent dyes 6-CFDA and propidium iodide (PI).

PA6 murine stromal cells were maintained in F-MEM medium containing minimum essential medium α (MEMα), 10%FBS, 25 U/ml penicillin and 25 µg/ml streptomycin. Medium was changed every third day.

### Preparation of hESC extract

Briefly, 4×10^6^ hESCs were treated with 0.1 µg/ml collagenase IV and dispase. The resultant cells were washed twice with D-PBS and once with lysis buffer (50 mM NaCl, 5 mM MgCl_2_, 100 mM HEPES, 1 mM dithiothreitol (DTT), 0.1 mM phenylmethylsulfonyl fluoride (PMSF) and 0.1% protease inhibitor cocktail (Sigma)) by centrifuging at 400×g for 5 min. The same volume with that of the cell pellet of ice-cold cell lysis buffer was used to resuspend the cells in 200 µl aliquots and were incubated on ice for 45 min. Swelled ESCs were then subjected to sonication on ice with a LABSONIC® M ultrasonic homogenizer (Sartorius Stedim Biotech, France) at 30% amplitude and 0.4-sec pulse cycle for 60–90 sec or until complete disruption of the cells and nuclei was achieved by observation under the microscope. Cell lysates were centrifuged at 15,000×g for 15 min at 4°C and the supernatant was collected. The ESC extracts were then analyzed for osmolariity and protein concentration. Cell extract from same number of HFFs was prepared using the same protocol and was used as a control. Both hESC and HFF extracts used for reprogramming contained 30–35 mg/ml protein.

The toxicity of the extracts was assessed after extract preparation. This was done by incubating 50,000 HFFs in 30 µl extracts for 1 hour. Only cell extracts that did not induce apoptosis (as determined by intact cell morphology under microscope) was used for reprogramming.

### HFF membrane permeabilization and hESC extract treatment

HFFs were washed twice in ice-cold D-PBS followed by one time wash in ice-cold HBSS by centrifuging at 500×g for 10 min. Cell pellets were resuspended in appropriate volume of ice-cold HBSS to make a concentration of 500,000 cells per reaction in each 1.5 ml microcentrifuge tube on ice. Following centrifugation at 500×g for 5 min at 4°C, cells were resuspended in ice-cold HBSS with 350 ng/ml SLO and incubated for 2 min in a 37°C water bath (50 µg/ml 10,000 M_r_ Texas red-conjugated dextran was used to evaluate the efficiency of SLO treatment). The cells were then incubated in a 37°C incubator horizontally for 50 min with occasional tapping to maintain cells in suspension. After permeabilization, cells were resuspended in 300 µl ESC extract containing 1 mM ATP, 100 µM GTP, 25 µg/ml creatine kinase, 10 mM phosphocreatine and 1 mM NTP mix followed by incubation at 37°C in a water bath for 1 h, with occasional tapping.

After treatment, mTeSR™1 medium (StemCell Technologies) supplemented with 2 mM CaCl_2_ was added to the cells and the cells were transferred to Matrigel (BD Biosciences)-coated cell culture plates for 2–4 h. After removing floating cells and medium, fresh mTeSR™1 medium was added and the medium was changed on a daily basis.

### Directed differentiation of the reprogrammed cells into neuronal cells

Differentiation experiments were designed according to previous published protocols [49], [50]. Briefly, PA6 cells were plated in tissue cultured plates coated with 0.1% Gelatin. After reprogramming treatment and when the stem cell-like colonies had been of reasonable sizes, the colonies were physically dissected and the clumps were broken by pipetting. The cells were then added to tissue culture plates coated with PA6 cell monolayer in differentiation medium containing MEMα, 10% SR, 1 mM sodium pyruvate, 0.1 mM non-essential amino acid and 0.1 mM 2-mercaptoethanol. hESCs were used as a positive control. Differentiation medium was changed on day 4 and every second day thereafter, until day 28.

### Immunofluorescent staining

Cells were collected and fixed in 4% paraformaldehyde and seeded onto poly-L-lysine coated slides (Menzel-Glaser) and permeabilised with 0.2% v/v Triton-X-100 followed by blocking with 10% normal serum in 3% bovine serum albumin (BSA) in PBS. Relevant primary and secondary antibodies in 1% BSA were then applied. Antibodies used included: Rabbit polyclonal to OCT4 (Abcam), Rabbit polyclonal to NANOG (Abcam), Goat polyclonal to LAMIN A/C (Santa Cruz Biotechnology), Mouse monoclonal to 5-Methyl Cytidine (Abcam), Rabbit polyclonal to Acetyl-Histone H3 (Lys9) (Cell signaling), Rabbit polyclonal to PAX6 (Abcam), Rabbit polyclonal to SOX1 (Abcam), Mouse monoclonal to NESTIN (Millipore), Rabbit polyclonal to TUJ (Covance), Mouse monoclonal to MAP2 (Covance), Mouse monoclonal to TH (R&D systems), Goat anti-Mouse IgG (FITC) (Millipore), Goat anti- Rabbit IgG (FITC), Abcam, Rabbit anti-Goat IgG (FITC) (Millipore), Goat anti-Rabbit IgG-H+L (Alexa Fluor 594) (Invitrogen). Nuclei were stained with Prolong gold anti-fade reagent with DAPI (Molecular probes, Invitrogen) before being observed using a fluorescent microscope (Leica DMI3000).

### Immunoblotting analysis

The cells were collected and lysed in RIPA buffer and the proteins were separated via SDS-PAGE. Briefly, 2 µg of protein were loaded onto 10% Tris-HCl gel (Biorad) and electrophoresis was performed at 140 V for 40 min. The proteins were then transferred to nitrocellulose membranes and blocked with 10% normal serum in 3% BSA. This was followed by probing with primary antibodies at 4°C overnight and secondary antibodies at room temperature for 1 h. enhanced chemiluminescence (ECL) kit (Amersham) and X-ray film (Hyperfilm ECL, Amersham) were used to detect cross reactive proteins and standard developing and fixing reagents (Kodak) was used to develop the exposed film. Antibodies applied were Rabbit polyclonal to OCT4 (Abcam), Rabbit polyclonal to NANOG (Abcam), Goat polyclonal to LAMIN A/C (Santa Cruz Biotechnology), Sheep polyclonal to 5-Methyl Cytosine (Novus Bio), Rabbit polyclonal to Acetyl-Histone H3 (Lys9) (Cell signaling), Rabbit anti-goat IgG HRP (CALBIOCHEM), Goat anti-Mouse IgG HRP (Millipore), Goat anti-Rabbit IgG HRP (Abcam), Rabbit anti-sheep IgG HRP, (Abcam).

### RT-PCR analysis

Total RNA was extracted from whole cells using the Illustra RNAspin Mini RNA Isolation Kit (GE healthcare). cDNA was generated by standard reverse transcription using SuperScript III First-Strand Synthesis System and Oligo (dT) primers (Invitrogen). PCR was performed using Hybaid PCR express Thermocycler. The PCR reaction mix contained the following components: 2 µl 10× PCR Buffer (−Mg^2+^), 0.6 µl 50 mM MgCl_2_, 0.4 µl 10 mM dNTPs, 0.4 µl forward primer, 0.4 µl reverse primer, 0.2 µl Taq DNA Polymerase and 15 µl PCR water to make a final reaction volume of 20 µl. The reaction conditions were: 94°C for 2 min, followed by 95°C for 30 sec, 59°C for 30 sec, 72°C for 30 sec (35 ceyles), then 72°C for 5 min. Primer pairs were: *PAX6* forward TCAGCACCAGTGTCTACCAACCAA, reverse ATCATAACTCCGCCCATTCACCGA; *SOX1* forward CAATGCGGGGAGGAGAAGTC, reverse CTCTGGACCAAACTGTGGCG; *NESTIN* forward GGCAGCGTTGGAACAGAGGTTG, reverse CTCTAAACTGGAGTGGTCAGGGCT; *TUJ* forward ACAACGAGGCCTCTTCTCACAAGT, reverse TTTCACACTCCTTCCGCACCACAT; *LMX1b* forward AACTGTACTGCAAACAAGACTACC, reverse TTCATGTCCCCATCTTCATCCTC; *DAT* forward AACTCCCAGTGTGCCCATGAGTAA, reverse AGCCAATGACGGACAGGAGAAAGT; *TH* forward GTCCCCTGGTTCCCAAGAAAAGT, reverse TCCAGCTGGGGGATATTGTCTTC; *β-ACTIN* forward ACGGCATCGTCACCAACT, reverse AGGAAGGAAGGCTGGAAGAG.

### Bisulfite treatment and bisulfite sequencing

DNA was isolated using the DNeasy Blood & Tissue Kit (Qiagen) and then treated with the EZ DNA Methylation-Gold Kit™ (Zymo Research) for bisulfite conversion. Converted DNA was amplified by PCR using the primers listed below. The annealing temperatures were 64°C for *OCT4*, 57°C for *C-MYC*, 59°C for *SOX2* and 63°C for *NANOG* and *KLF4*. The PCR products were then cloned into the pGEM-T Easy Vector (Promega) system for sequencing. Primer pairs used were: *OCT4* forward: TTGGGGGTTGGGTTAGGTTTTGAG, reverse: CTCCAACTTCTCCTTCTCCAACTTC; *NANOG* forward: TTGTTGTTTAGGTTGGAGTATAGTGG; reverse: ATAACCCACCCCTATAATCCCAATAA; *SOX2* forward: GATGGTTTAGGAGAATTTTAAGATG, reverse: CRTAACTATCCATACRCTAATTCAC; *KLF4* forward: GGATTTTTTGTTATAGAGGAGGTTT, reverse: TCTCCTAAACCTAAACTTTATTCTC; *c-MYC* forward: GTAAATAGGAGGAGGGTTGATGYG, reverse: CATCCAAATTAAACCACTAAACTC.

### DNA finger printing

When ESC-like colonies could be identified, they were manually dissected and DNA extracted using the DNeasy Blood & Tissue Kit (Qiagen). hESC and HFF DNA was extracted at the same time. 5–10 µl of DNA at 20–30 ng/µl for each sample were sent to DNA Labs, Sydney IVF (Sydney, Australia) and DNA profiling using the internationally recognized Identifiler system was performed, followed by short tandem repeat (STR) analysis.

### qPCR

Total RNA was extracted from whole cells using the Illustra RNAspin Mini RNA Isolation Kit (GE healthcare). cDNA was generated by standard reverse transcription using SuperScript III First-Strand Synthesis System and Oligo (dT) primers (Invitrogen). qPCR was performed on a RoterGene 3000 real-time PCR machine. The reaction comprised 10 µl SensiMixPlus SYBR, 0.5 µl forward and reverse primer, 2 µl diluted DNA template and 7 µl PCR water. The reaction conditions were 95°C for 10 min, followed by 40 cycles of 95°C for 15 sec, 60°C for 30 sec and 72°C for 20 sec. The relative expression of genes were normalized against a house keeping gene GAPDH. Primer pairs were: *OCT4* forward: TGGGCTCGAGAAGGATGTG, reverse: GCATAGTCGCTGCTTGATCG; *NANOG* forward: AGAAGGCCTCAGCACCTAC, reverse: GGCCTGATTGTTCCAGGATT; *SOX2* forward: ATGCACAACTCGGAGATCAG, reverse: TATAATCCGGGTGCTCCTTC; *KLF4* forward: CCCAATTACCCATCCTTCCT, reverse: ACGATCGTCTTCCCCTCTTT; *c-MYC* forward: CTGGTGCTCCATGAGGAGAC, reverse: CTCTGACCTTTTGCCAGGAG; *TP53* forward; ACCACCATCCACTACAACTACAT, reverse: ACAAACACGCACCTCAAAGC; *CDKN1A* forward: TGATTAGCAGCGGAACAAG, reverse: AAACAGTCCAGGCCAGTATG; *BCL2* forward: GCTCTAAAATCCATCCAG, reverse: CCTCTCCATCATCAACTT; *HDM2* forward: GGCTTTGATGTTCCTGATTG, reverse: CTTTGTCTTGGGTTTCTTCC; *CASP3* forward: TTTGAGCCTGAGCAGAGACA, reverse: CGTATGGAGAAATGGGCTGT; *CASP9* forward: CTAGTTTGCCCACACCCAGT, reverse: GGGACTGCAGGTCTTCAGAG; *GADPH* forward: CATCCCTTCTCCCCACACAC, reverse: AGTCCCAGGGCTTTGATTTG.

### Statistical analysis

Data presented as mean ± SD from three independent experiments. The statistics generated in this study were performed using GraphPad Prism 5 (GraphPad Software, Inc). Significant difference was analyzed by one-way ANOVA or two-way ANOVA followed by Tukey-Kramer's tests. The results were considered significant when P values were less than 0.05.

## Supporting Information

Table S1 Cell growth rate and viability of HFFs after treatment with different concentrations of 5-aza-dC, TSA and ATRA.
(0.66 MB TIF)

Figure S1 Immunofluorescent analysis of NANOG expression in the cells. (a) hESCs, (b) HFFs, (c) HFF extract-treated HFFs and (d) hESC extract-treated HFFs.
(1.20 MB TIF)

Figure S2 Immunofluorescent analysis of global 5-methyl cytosine level in the cells. (a) hESCs, (b) HFFs, (c) HFF extract-treated and (d) hESC extract-treated HFFs.
(1.18 MB TIF)

Figure S3 Bisulfite sequencing of CpG islands of SOX2, KLF4 and c-MYC.
(1.39 MB TIF)

Figure S4 Immunofluorescent analysis of global 5-methyl cytosine level in the cells. (a) hESCs, (b) HFFs, (c) HFFs after DNMT/HDAC inhibitor treatment and (d) HFFs after combined treatment.
(1.17 MB TIF)
