# Supplementary figures and images for: A Combined Epigenetic and Non-Genetic Approach for Reprogramming Human Somatic Cells
Source: PLoS One. 2010 Aug 19;5(8):e12297. doi: 10.1371/journal.pone.0012297 (PMC2924394; doi:10.1371/journal.pone.0012297)

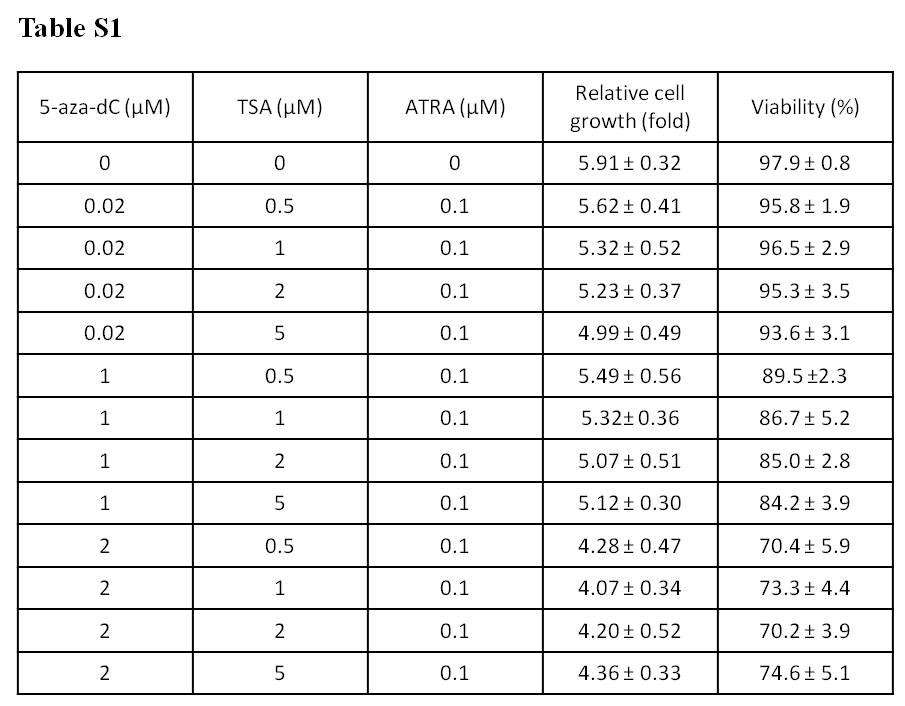

Supplement: Table S1 — Cell growth rate and viability of HFFs after treatment with different concentrations of 5-aza-dC, TSA and ATRA. (0.66 MB TIF) [file pone.0012297.s001.tif]

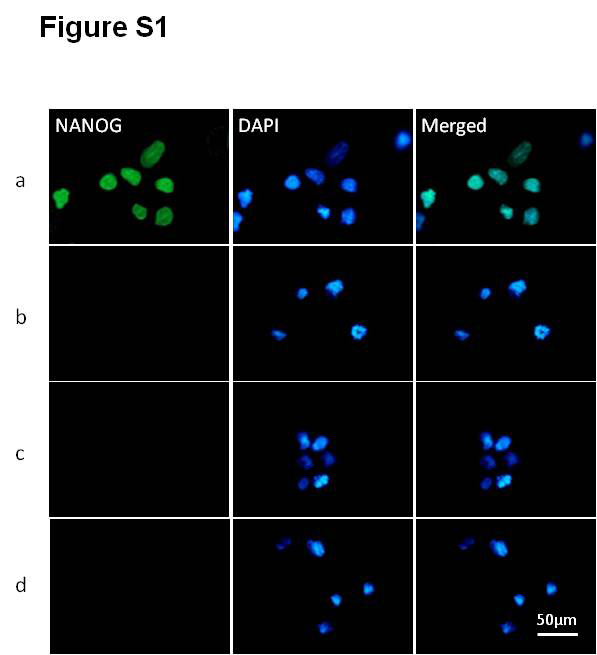

Supplement: Figure S1 — Immunofluorescent analysis of NANOG expression in the cells. (a) hESCs, (b) HFFs, (c) HFF extract-treated HFFs and (d) hESC extract-treated HFFs. (1.20 MB TIF) [file pone.0012297.s002.tif]

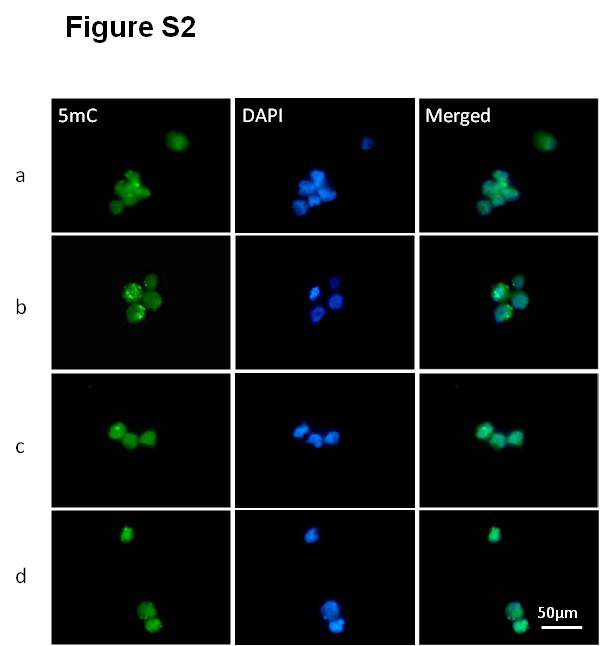

Supplement: Figure S2 — Immunofluorescent analysis of global 5-methyl cytosine level in the cells. (a) hESCs, (b) HFFs, (c) HFF extract-treated and (d) hESC extract-treated HFFs. (1.18 MB TIF) [file pone.0012297.s003.tif]

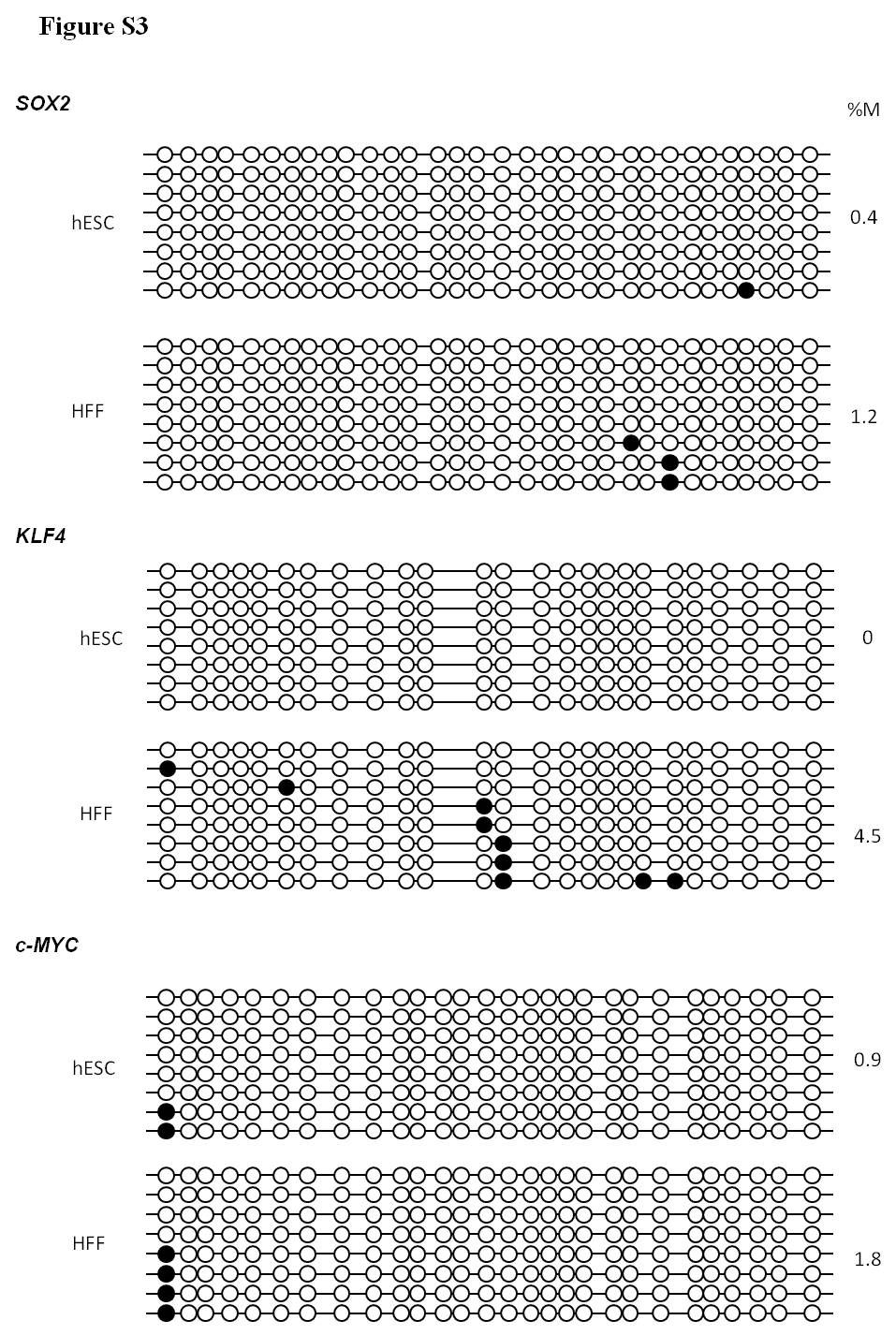

Supplement: Figure S3 — Bisulfite sequencing of CpG islands of SOX2, KLF4 and c-MYC. (1.39 MB TIF) [file pone.0012297.s004.tif]

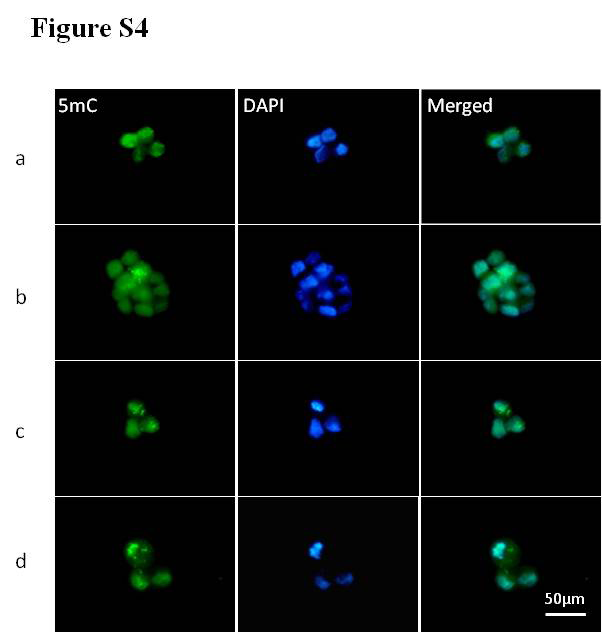

Supplement: Figure S4 — Immunofluorescent analysis of global 5-methyl cytosine level in the cells. (a) hESCs, (b) HFFs, (c) HFFs after DNMT/HDAC inhibitor treatment and (d) HFFs after combined treatment. (1.17 MB TIF) [file pone.0012297.s005.tif]
